# Macrophage activation of the TREM2-DAP12-SYK pathway shapes the adipose tissue microenvironment in obesity and unveils the therapeutic potential of natural compounds egcg and SMRR

**DOI:** 10.3389/fimmu.2025.1694985

**Published:** 2025-12-11

**Authors:** Wang Luoying, Yi Xingcheng, Cong Donghang, Long Mengtuan, Wang Ping, Zhang Lijuan, Li Yupeng, Zhao Tianyi, Chen Hongyu, Wang Song, Zang Zijun, Zheng Hanyu, Chen Shengchao, Fu Cong, Su Xiaoyun

**Affiliations:** 1 Department of Regenerative Medicine, School of Pharmaceutical Sciences, Jilin University, Changchun, China; 2 Laboratory of Cancer Precision Medicine, First Hospital of Jilin University, Changchun, China; 3 Department of Cadre Ward, The First Hospital of Jilin University, Changchun, China; 4 Department of Otolaryngology-Head and Neck Surgery, First Hospital of Jilin University, Changchun, China; 5 Jilin Medical University, Jilin, China; 6 School of Software, Tsinghua University, Beijing, China; 7 Institute of Changbai Mountain Biology Germplasm Resources, Tonghua Normal University, Tonghua Jilin, China; 8 School of Computer Science, Hangzhou Dianzi University, Hangzhou, China; 9 Key Laboratory of Organ Regeneration and Transplantation of the Ministry of Education, The First Hospital of Jilin University, Changchun, Jilin, China

**Keywords:** obesity, macrophages, single-cell gene regulatory networks, targeted drugscreening, adipose tissue microenvironment

## Abstract

Obesity is a major global health burden, with current therapies limited by metabolic adaptation and adverse effects. Although transcriptomic studies reveal widespread gene alterations in obesity, key drivers and their cell-specific origins in adipose tissue remain unclear. Defining these regulators is critical for understanding immune-metabolic imbalance and developing targeted interventions. We integrated bulk transcriptomics (n=434) with single-cell RNA-seq (194,608 cells from 24 adipose samples) to identify BMI-associated gene modules and macrophage regulatory programs. Cell-specific networks, subtype-specific gene regulatory networks, pseudotime trajectories, and cell–cell communication analyses delineated macrophage heterogeneity. Molecular docking assessed interactions between candidate drugs and the TREM2–DAP12–SYK pathway, and *in vivo* studies evaluated the therapeutic potential of EGCG and SMRR in high-fat diet mice. Our analyses revealed significant molecular and microenvironmental differences between healthy and obese adipose tissue. Eight BMI-associated genes—SYK, CD86, CSF1R, HCK, TYROBP, LAPTM5, ITGB2, and ACTB—were predominantly expressed in macrophages. Single-cell profiling identified macrophage subtypes (C4, C6, C10) with distinct regulatory roles in adipocyte communication. Dysfunction of the TREM2–DAP12–SYK axis underpinned obesity-associated macrophage states, while EGCG and SMRR reactivated this pathway, mitigating obesity and metabolic dysfunction *in vivo*. These findings define a macrophage-centered regulatory network driving obesity progression and highlight actionable therapeutic targets.

## 1 Introduction

The global epidemic of obesity is rapidly gaining prominence, driven by shifts in dietary habits and lifestyle choices, resulting in a dramatic surge in the prevalence of obesity worldwide. This burgeoning crisis is intricately linked with the rise in incidences of type 2 diabetes, cardiovascular ailments, and specific types of cancer, thereby presenting a formidable public health dilemma on a global scale (1). While healthy dietary habits and physical activity remain primary interventions, the phenomenon of adaptive feedback regulation often leads to a plateau in the weight loss process, rendering sustained weight reduction a daunting challenge (2). Existing drug treatments such as GLP-1 receptor agonists have shown some efficacy; however, they are often accompanied by long-term gastrointestinal side effects (3). Therefore, identifying new therapeutic targets and drugs to remodel the adipose tissue microenvironment (ATM) in obesity and reduce treatment adverse effects is a key challenge in current obesity research.

As advancements in high-throughput sequencing technologies persist, next generation sequencing and single-cell RNA sequencing (scRNA-seq) are increasingly applied in the field of obesity research (4). Presently, numerous researchers are endeavoring to unravel the heterogeneity present in obesity samples across various organs at the transcriptional level, aiming to pinpoint diagnostic biomarkers or novel targets of obesity treatment. Gusev et al. conducted a transcriptome-wide association scan (TWAS) using expression data from blood and adipose tissue samples collected from 3,000 obese individuals. They identified 69 genes significantly associated with changes in body mass index (BMI) and lipid levels (5). Moreover, Researchers are studying adipose tissue macrophages at the single-cell level to develop strategies for enhancing obesity management. Hildreth et al. performed scRNA-seq on the stromal vascular fraction of white adipose tissue (WAT) from healthy and obese individuals. They identified distinct inflammatory interactions and enriched signaling clusters involving immune cells in WAT (6).

While significant progress has been made in identifying essential therapeutic and diagnostic targets, as well as specific cell subtypes, at the transcriptomic and single-cell levels, understanding the regulatory patterns of these crucial therapeutic targets within ATM remains elusive. Thus, a significant research challenge is identifying therapeutic targets and understanding their interactions among diverse cellular components within ATM. This effort seeks to discover new intervention methods and implement combined treatment strategies. In recent years, immune microenvironment regulation has found widespread application across various fields, including cancer treatment and anti-aging efforts (7, 8), Macrophages are essential components of the immune microenvironment, crucial for maintaining immune homeostasis and regulating immune-inflammatory processe (9). Zhao et al. demonstrated that adipose-derived stem cells can induce M2 macrophage polarization and reduce inflammation, potentially converting WAT into beige adipose tissue through exosome release (10). The composition and differentiation stages of macrophage subtypes may significantly impact susceptibility to obesity or resilience against it.

**Figure 1:**
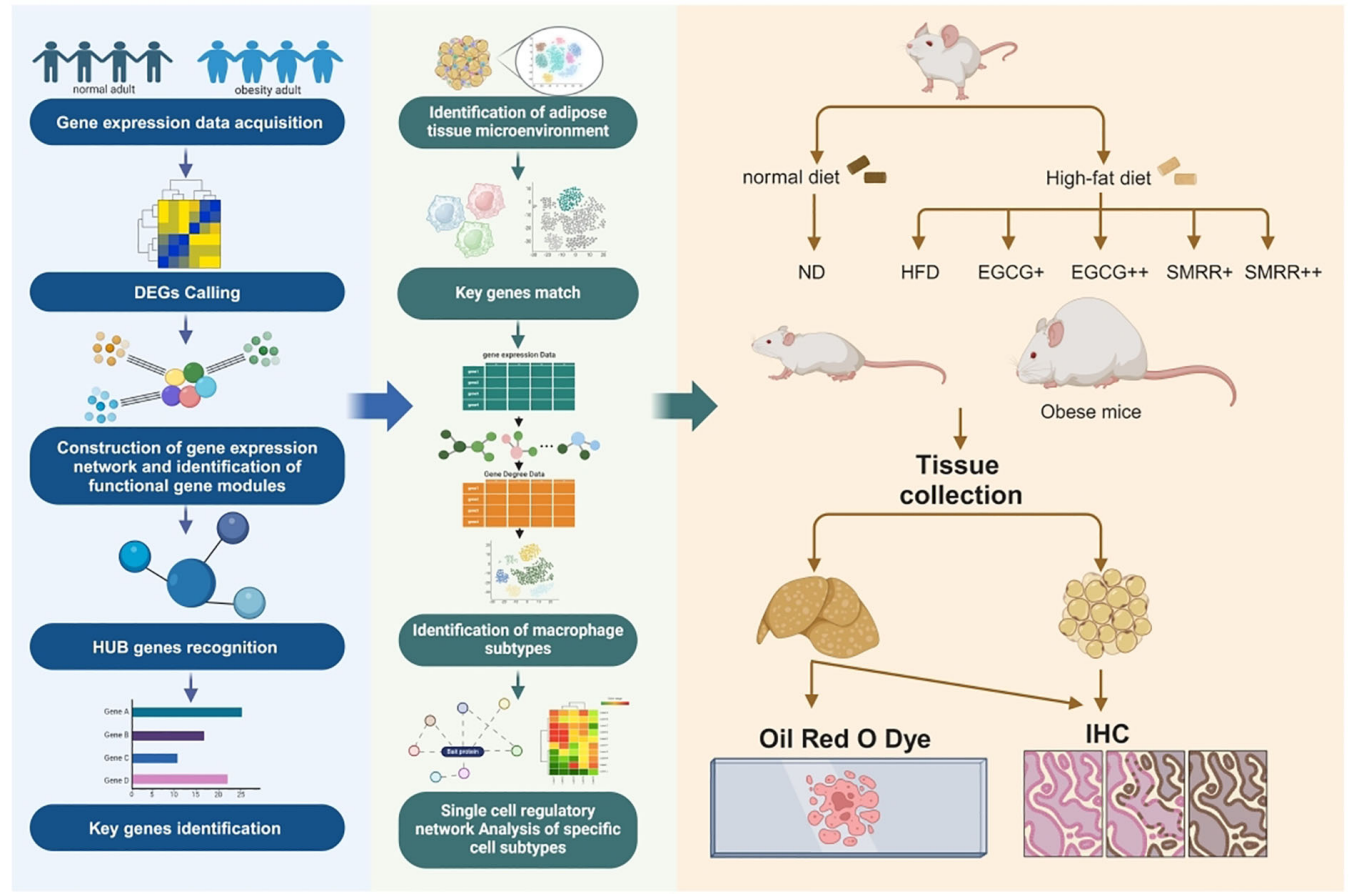
The workflow of this study. Step a Obtaining gene expression profiles in healthy and obese populations. Step b calling DEGs between healthy and obese populations. Step c Identifying functional gene modules using a Multilevel combined with k-means algorithm. Step d Identifying HUB genes based on key module M2. Step e Key genes identification by randomForest algorithm. Step f Constructing single-cell landscape of ATM in healthy and obese populations using the Seurat standard pipline. Step g The localization of key genes in macrophages. Step h Identifying macrophage subgroups based on NCM. Step i Functional analysis of specific macrophage subtypes. Step j Validation through in vitro experiments and functional verification of targeted DAP12-SYK pathway drugs SMRR and EGCG.

To explore pivotal therapeutic targets within obese samples and elucidate their regulatory mechanisms within the ATM, we applied a gene co-expression network analysis method (11, 12) to pinpoint gene modules and key genes intricately associated with BMI changes. Subsequently, we matched these key genes onto a scRNA-seq dataset comprising adipose tissue samples at different BMI levels. These key genes were found predominantly expressed in macrophages, implying a close association between alterations of adipose tissue macrophages and obesity. Further, we developed a single-cell gene regulatory network analysis strategy, and systematically elucidated the transitions of macrophage phenotypes from healthy to severely obese individuals. This provided insights into heterogeneity, functional specificity, and cellular communication modes between macrophage subtypes and adipocytes during different disease progressions. Fortunately, we have found significant differential expression of the DAP12 signaling pathway in specific macrophages subtype C4, C6, C10 across different obesity levels. Finally, we validated above findings *in vivo* and discovered that epigallocatechin-3-gallate (EGCG) and Salviae miltiorrhizae Radixet Rhizoma (SMRR) inhibits obesity through aforementioned pathway (**Figure 1**). This study provides compelling evidence for deeper understanding of the molecular mechanisms of obesity and potential therapeutic strategies.

## 2 Materials and methods

### 2.1 Transcriptome data preprocessing in different BMI populations

In this study, we selected gene expression data (GSE135134) from subcutaneous adipose tissue samples of 434 participants. Genes with zero expression in the gene expression profile were first filtered out to ensure robust analysis. Subsequently, the raw count data was normalized to Counts Per Million (CPM) for further analysis using the EdgeR package in R version 3.28.1. It’s noteworthy that this study is based on publicly available data ethical approved.

### 2.2 Identification of differentially expressed genes and construction of the gene co-expression network in obese adipose tissue

To investigate gene expression disparities between adipose tissues in healthy and obese groups, we defined normal body weight as BMI ≤ 25 kg/m2 and BMI ≥ 25 kg/m2 as obese (13), and got 379 obese samples(expreimental group) and 55 healthy samples(negative control group). Using predefined thresholds(|logFC| > 0.25 & P < 0.05), we applied the T-test algorithm to identify differentially expressed genes (DEGs) for further analysis.

The Pearson correlation coefficient (PCC) was utilized as a pivotal metric for assessing synergistic or antagonistic interactions between genes (14). Correlation coefficient between gene pairs within the DEGs was calculated using the PCC algorithm, thus constructing a gene co-expression network (|PCC| > 0.65 & P < 0.05). Ultimately, the largest connected subgraph including all co-expression relationships was defined as the gene co-expression network in obese population.

### 2.3 Identification of functional gene modules in obese adipose tissue

To unravel functional gene modules in obese population, we initiated our analysis by leveraging the previously established gene co-expression network as background data. We applied a Multilevel algorithm to identify gene communities with similar topological structures, and only kept those with more than 20 genes for further analysis.

Principal Component Analysis (PCA) was applied to reduce the dimensionality of gene expression data within each gene community and identified the first principal component for each module termed as the Community Feature Vectors (CFVs). Take the similarity in gene expression within communities under consideration, we re-clustered the gene communities and defined functional gene modules using CFVs as centroids and PCC as the distance metric based on the Kmeans algorithm.

Multilevel algorithm is implemented via the function multilevel.community() in the igraph package in R version 4.2.2 (15), and PCA algorithm is conducted using the function prcomp() in R version 4.2.2 (16).

### 2.4 Functional gene module association analysis related to BMI

To explore the connection between the aforementioned functional gene modules and BMI changes, we applied the Spearman correlation algorithm to calculate the correlation between the first principal component and BMI in each functional gene module, and constructed a correlation matrix. Ultimately, we identified the functional gene module with the strongest BMI association as the key gene module.

### 2.5 Localization analysis of key gene modules on chromosomes

For the analysis of the chromosomal positioning of the key gene module, we utilized the Rideogram tool to map the key gene module onto the human reference genome (hg19) to obtain chromosomal location results.

The Rideogram tool achieves this through the function ideogram () in the R package Rideogram.

### 2.6 Construction of protein-protein interaction network of key gene modules and identification of key genes

To uncover hub genes within the key gene module, we started by retrieving the Protein-Protein Interaction (PPI) network of the key gene module from the STRING database (http://string-db.org) (17). The centrality of genes within this network was calculated by PageRank algorithm. The top 25 genes with the highest scores were identified as hub genes. Finally, we employed the Random Forest algorithm for feature selection of hub genes, and selected genes that meet appropriate threshold as key genes (IncMSE>5.3 & IncNodePurity >2.9).

PageRank algorithm is implemented via the function page_rank () in the igraph package, and the random Forest algorithm through the function randomForest () in the randomForest package.

### 2.7 scRNA-Seq data acquisition and preprocessing of adipose tissue across varied BMI

We downloaded a scRNA-seq dataset comprising 24 samples with varying BMIs from GEO dataset (GSM5359325~GSM5359337, GSM5820679~GSM5820689). Refering to the obesity classification standards outlined by the World Health Organization (WHO), we devided 24 samples into 3 distinct groups: 7 healthy-individual samples (HD) (BMI < 25 kg/m²), 5 moderately obese (MO) samples (30 kg/m² < BMI < 37.5 kg/m²), and 12 severely obese (SO) samples (BMI > 37.5 kg/m²) (18).

The gene expression matrix was analyzed by R software (version 4.3.1) with the Seurat package (4.3.0.1). Low quality cells were removed if they met three criteria: (1) < 500 unique molecular identifiers (UMI) or >3500 UMIs; (2) < 200 genes; (3) >10% UMIs derived from the mitochondrial genes. After the removal of the lower quality cells, a total of 194,608 cells were kept for further analyses. Then, UMI counts were normalized by the function NormalizeData. Top 5000 genes with the highest entropy scores were defined as highly variable feature genes (19). Highly variable feature genes were calculated using Intrinsic entropy model. Finally, the function ScaleData was conducted to scale and center highly variable feature genes in the datasets.

The Intrinsic entropy model analysis was conducted using the Get_entropy () function in the IEntropy package.

### 2.8 Cell type annotation in the ATM

Based on the aforementioned scale data, we employed the PCA for feature extracting. Top 10 principal components were calculated to reveal the main axes of variation and denoise the data. Cells were clustered by Shared Nearest Neighbor (SNN) algorithm using the expression profiles. For visualization, UMAP dimensionality reduction were applied by using RunUMAP function. Finally, SingleR algorithm nd known cell markers were annotated cell types of clusters.

Specifically, the function RunPCA was used to implement PCA algorithm, function FindClusters for SNN algorithm, and SingleR algorithm was executed through the function SingleR. HumanPrimaryCellAtlasData dataset was selected as the reference, with known gene markers listed in **Supplementary Table S1**.

### 2.9 Macrophage single-cell gene regulatory networks construction

To explore the cellular regulatory patterns in macrophages, we utilized macrophage expression data from the aforementioned samples and constructed single-cell gene regulatory networks of each cell using the Cell Specific Network (CSN) algorithm (20).

Specifically, the regulatory relationship between genes was calculated using the CSN algorithm, formula is as follows:


$$\rho_{xy}=\frac{n_{xy}^{\left(k\right)}}{n}-\frac{n_{x}^{\left(k\right)}}{n}\cdot \frac{n_{y}^{\left(k\right)}}{n}$$

In the equation, x represents gene x, y represents gene y, k represents cell k, and n represents the total number of cells within the region under consideration.

### 2.10 Macrophage single cell gene regulatory network node feature extraction

The degree of each node in Macrophage single cell gene regulatory network was counted as its attribute. For a given node v, where N(v) represents the set of its neighbors within the network:


Degree(v)=|N(v)|

We pooled all genes in the single-cell gene regulatory network and constructed a degree vector d for each gene:


d=|d1,d2, di…,dn|

Here, n represents the number of cells, and $d_{i}$ was defined as the degree of certain gene in the single-cell gene regulatory network of the i-th cell.

Finally, we put all degree vectors of macrophages together and constructed a macrophage gene regulatory network characterization matrix (NCM).

### 2.11 Macrophage subtypes identification

To identify subclusters within Macrophages, NCM of macrophages were analyzed with normalizing, dimensionality reduction and clustering by SNN algorithm (resolution = 0.5).

### 2.12 Differential degree genes and featured degree gene identification

Based on NCM we constructed earlier, each macrophage subtype was treated as a experimental group, while the other subtypes served as the negative control group, differentially degree genes (DDGs) of each subtype were identified using WIlcoxon tests. Specifically, genes exhibiting logFC>0.25 and P <0.05 were classified as up-regulated DDGs, while those with logFC<0.25 and P <0.05 were categorized as down-regulated DDGs.

ROC curves were used to identify featured degree genes (FDGs) which exhibited robust discriminatory power compared to other subtypes, and screening threshold was set as logFC > 0.5 & AUC > 0.75.

The Wilcoxon test and AUC value calculations were implemented using the function FindAllMarkers.

### 2.13 Macrophage differentiation patterns analysis

To investigate the differentiation patterns of macrophages in adipose tissues of varying BMI, we applied Monocle (version 2.28.0) to NCM (21). Temporal differential genes (TDGs) are defined as the significant genes (q < 0.05, top 3000 genes) in the studied cells by using the function differentialGeneTest. Based on these TDGs, cell differentiation trajectories are constructed using default parameters in Monocle through dimensionality reduction and cell ordering.

### 2.14 Distinct BMI-specific macrophage subtype-specific gene regulatory network construction and gene module identification

Based on the single-cell gene regulatory networks of macrophage subtype C4, C6, C10, Driver genes for each subtype were defined as those genes present in at least one connection with other genes in 50% of the cells of each subtype. Furthermore, the subtype-specific gene regulatory network (ssGRN) was defined as the collection of regulatory relationships existing among driver genes in 50% of the cells of that subtype.

To reveal the functional gene modules within the ssGRNs, we applied the Deepwalk algorithm to construct embedding vectors for each gene within the ssGRN. Then, we calculated the embedding similarity between genes using cosine similarity, thereby constructing a gene similarity adjacency matrix. Finally, we utilized a Gaussian Mixture model clustering algorithm to identify gene modules. For each gene module, GO enrichment analysis was conducted, and top 60 GO terms ranked by ascending P value were integrated. Lastly, we defined the highest -log P value of each term across all gene modules as an indicator of the enrichment level of that term within the subtypes.

### 2.15 Adipocyte subtypes identification and cell communication with macrophages

To identify subclusters within adipocytes, the cells belonging to adipocytes were re-analyzed separately with normalization, dimensionality reduction, and clustering by SNN algorithm. Ultimately obtained 26 subtypes of adipocytes (resolution = 0.5).

Subsequently, the iTalk algorithm was utilized to quantify cell communication between the 26 adipocyte subtypes and macrophage subtypes C4, C6, and C10. Significant ligand-receptor pairs were extracted from the communication, comprising the top 20 predicted pairs with P < 0.05 and avg (FPKM) > 1.

### 2.16 Animal experiment

#### 2.16.1 Drugs

EGCG (purity >98%) was obtained from Shanghai yuanye Bio-technology Co., Ltd. (Shanghai, China), SMRR (Tanshinone >30%) obtained from Shaanxi Hengling Natural Bioproducts Co., Ltd (Shaanxi, China).

#### 2.16.2 Animals and treatment

Male C57BL/6J mice (6–7 weeks, 18-22g) were acquired from SiPeifu (Beijing) Biotechnology Co., Ltd (Beijing,China). The mice were randomly divided into six groups (normal diet (ND), high-fat diet (HFD), low-dose EGCG group, high-dose EGCG group, low-dose SMRR group, high-dose SMRR group, n=8 per group). The ND group was fed ND, while the remaining groups were fed HFD. After three weeks on their respective diets, the treatment groups were intragastrical administered the following dosages: the low-dose SMRR group received SMRR (400mg/kg), the high-dose SMRR group received SMRR (800mg/kg), the low-dose EGCG group received EGCG (40mg/kg), and the high-dose EGCG group received EGCG (80mg/kg) for 8 weeks. The mice in the ND and HFD groups were fed ND or HFD and intragastrically administered distilled water (10ml/kg) for 8 weeks. At the end of the experiment, the mice were fasted overnight, followed by weight measurement and finally euthanasia. Livers and subcutaneous adipose tissue were collected, recored the adipose weight, portions of liver and adipose tissues were washed twice with sterile phosphate-buffered saline (PBS), then fixed in 4% paraformaldehyde overnight for further experiments. Additionally, part of the liver tissue was immediately snap-frozen in liquid nitrogen, and stored at −80°C for subsequent analysis.

All animal experiments were conducted in accordance with the guidelines approved by the Institutional Animal Care and Use Committee (IACUC) of Jilin univerisity School of Pharmaceutical Sciences (Approval No. 20210055) and complied with national regulations and international standards for the ethical use of laboratory animals. Mice were first anesthetized in an induction chamber with 4–5% isoflurane for 1–2 minutes to minimize pain and distress. Euthanasia was then performed by transferring the animals to a chamber filled with carbon dioxide at a flow rate of 30% of the chamber volume per minute, and exposure was maintained for at least 1 minute after respiration ceased. Death was further confirmed by cervical dislocation to ensure brainstem transection. All procedures were performed by trained personnel to minimize animal suffering and ensure ethical compliance.

### 2.17 Immunohistochemical staining

Subcutaneous adipose and liver were embedded in paraffin, sectioned at 4 μm and stained with hematoxylin and eosin (H&E). For immunohistochemical staining, The tissue sections were dewaxed and rehydrated, washed with 0.01 mol·L-1 PBS, and heated in 0.01 mol·L-1 citrate buffer (pH=6.0) for 10 minutes for antigen retrieval. After washing with PBS, 3% hydrogen peroxide was added to incubate for 15–20 minutes to block endogenous peroxidase activity. The sections were blocked with goat serum for 15–20 minutes, then incubated overnight at 4°C with antibodies TREM2 (A10482, 1:1000, Abclonal, China), DAP12(A14794, 1:1000, ABclonal, China), and SYK (382925, 1:1000, Zenbio, China). Biotinylated secondary IgG was added to the sections, incubated at room temperature for 15–20 minutes, followed by streptavidin-biotinylated horseradish peroxidase complex for 15–20 minutes, and DAB for color development, with hematoxylin counterstaining for 1 minute. Finally, images were acquired using a digital pathology slide scanner (Ultrafast Scanner 1.8, Philips), and the average optical density (AOD=integral optical density/image area) was calculated using ImageJ software to assess protein expression levels.

### 2.18 Oil red O staining

Frozen liver sections (8-10μm thick) were cut and affixed to slides, then fixed in 10% formalin solution for 10–15 minutes, and stained with Oil Red O solution (dissolved in 60% isopropanol) for 10–15 minutes at room temperature. Excess dye was gently washed off with 60% isopropanol. The sections were counterstained with hematoxylin for 10 seconds to 1 minute, rinsed with water, and mounted under a cover slip with glycerol gelatin in dark conditions. Observations were made under an Olympus BX50 microscope (Olympus, Tokyo, Japan).

### 2.19 Statistical analysis

All data are presented as mean ± SD. One-way analysis of variance (ANOVA) was used for intergroup differences, followed by the *post hoc* tests, with graphs generated using GraphPad Prism 8.4.0 software. P < 0.05 was considered statistically significant.

### 2.20 Molecular docking

The MOL2 structure files for epigallocatechin-3-gallate (EGCG) and SSMR were downloaded from the Traditional Chinese Medicine Systems Pharmacology Database (https://www.tcmsp-e.com/load_intro.php?id=43). Crystal structures of DAP12 (PDB ID: 4WOL), SYK (PDB ID: 1XBA), and TREM2 (PDB ID: 5ELI) were obtained from the (http://www.rcsb.org/). Molecular docking was performed using CB-Dock2 in PyMOL 2.3.0. The full protein structures were used as receptors, and docking poses were sorted based on their vina scores.

## 3 Results

### 3.1 Transcriptome data analysis reveals genetic changes in adipose tissue of obese individuals

**Figure 2:**
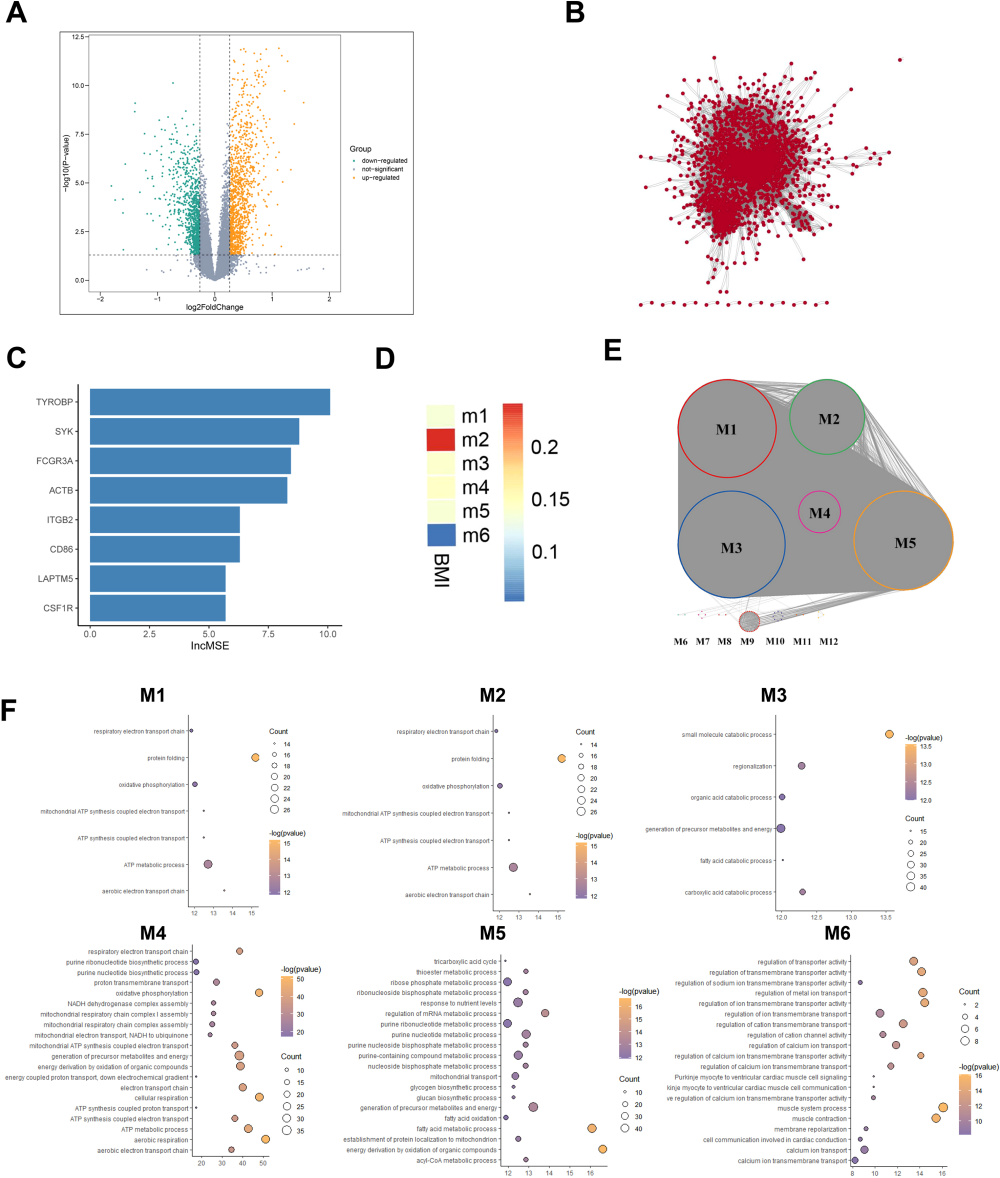
Obesity vs healthy population adipose tissue differential transcriptome atlas. **(A)** DEGs between Obese and Healthy groups. Yellow points represent upregulated DEGs, and green points represent downregulated DEGs. **(B)** Gene co-expression network based on the DEGs expression profile from the obese group. **(C)** Key Genes in Functional Gene Module M2 Using Random Forest Algorithm. **(D)** Association analysis between functional gene modules and BMI score, where blue indicates lower correlation and red indicates higher correlation. **(E)** Functional Gene Modules in the gene co-expression network. Different colors of nodes represent their membership in different gene modules. **(F)** GO enrichment analysis results of functional gene modules (M1~M6).

To identify gene expression differences between obese and health populations, we used healthy population as a reference and applied T-test with a threshold of |logFC| > 0.26 & P < 0.05 to identify DEGs, and got 1837 up-regulated genes (e.g., EGFL6, SDS, MMP9) and 2144 down-regulated genes (e.g., SUMF2, UBE2F, ASCC2) in the obese population (**Figure 2A**).

Given the pivotal role of gene interactions in executing biological functions, we focused on key gene modules influencing BMI alterations in the obese group. First, a gene co-expression network of DEGs in adipose tissue of obese individuals was constructed using PCC algorithm (**Figure 2B**), then we identified 6 functional gene modules using the Multilevel algorithm combining K means framework (**Figure 2C**). Module 2(M2) showed a highly positive correlation with BMI (**Figure 2E**). GO enrichment analysis revealed either highly specific and shared biological functions in 6 functional gene modules (**Figure 2F**). BMI, an effective obesity index widely used for assessing obesity in children, adolescents, and adults (22), is also applied in guiding adult obesity management and drug treatment (23). M2 participates in immune cell activation, such as T cells, as well as proliferation, migration, chemotaxis, and immune response activation, which indicated a close connection between obesity and abnormal immune system activation, all genes in M2 are predominantly situated on chromosome 19 (**Supplementary Figure S2A**), which is very intriguing. Chromosome 19 has been reported playing important role in many disease, and chromosome 19 mutations are prevalent in lung cancer as potential carcinogenic factors in malignant lung tumors, particularly non-small cell lung cancer (24). Among these M2 genes on Chromosome 19, CALM3 encoding calmodulin has garnered attention in Alzheimer’s disease (AD) research (25); APOE4 and APOC1A has been acknowledged as risk factors for late-onset AD (26). As mentioned above, abnormal alterations on chromosome 19 may indicate the co-occurrence of obesity and other diseases.

We constructed a PPI network using the STRING database and identified top 25 hub genes using the Pagerank algorithm (**Supplementary Figure S2B**). Additionally, by applying Random Forest feature selection algorithm to the network, 8 key genes were found strongly associated with BMI: SYK, CD86, CSF1R, HCK, TYROBP, LAPTM5, ITGB2, and ACTB (IncMSE>5.3 & IncNodePurity >2.9) (**Figure 2C**).

### 3.2 Single-cell level landscape construction of ATM changes during obesity

To explore the cellular heterogeneity of ATM during obesity progression, we analyzed scRNA-Seq data from 24 adipose tissue samples (7 HD, 5 MO, and 12 SO), totaling 194,608 cells. Using the standard Seurat pipeline, we identified cell types with SingleR and known cell markers (**Supplementary Figures S2C**, **S3A**). Our analysis revealed four distinct cell types in ATM: adipocytes, fibroblasts, macrophages, and T cells. Notably, T cells and macrophages emerged as the predominant resident immune cells in adipose tissue, potentially playing intricate roles in regulating adipocytes.

**Figure 3:**
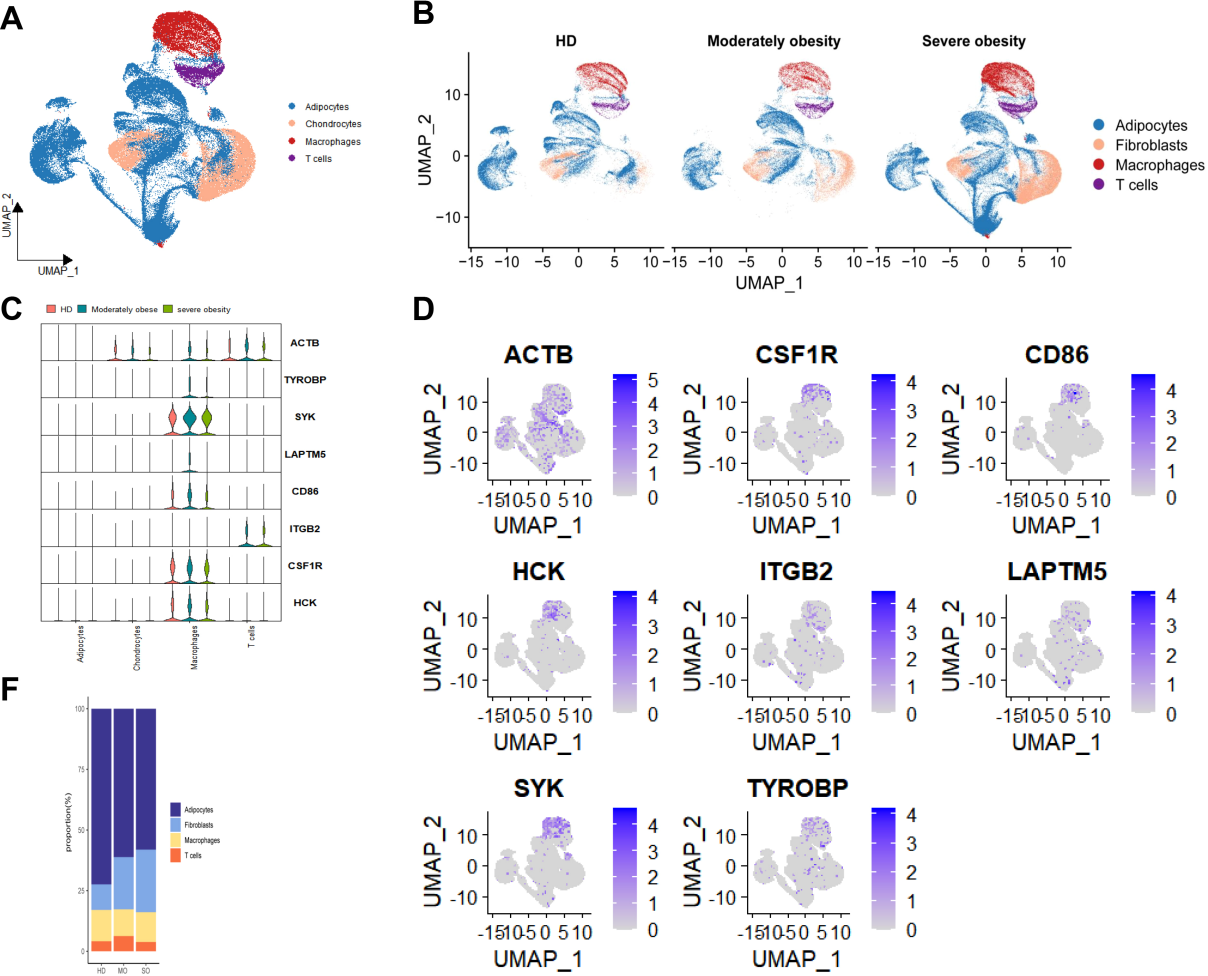
Panoramic landscape of ATM changes during obesity evolution map. **(A)** UMAP plot revealed Distribution of 4cell types of cells identified and color-coded. **(B)** UMAP plot revealed cellular heterogeneity with 4cell types of cells identified and color-coded. **(C)** Expression Distribution (violin plots) of 8 Key Genes (SYK, TYROBP, ACTB, etc.) in Each Cell Type of the HD, MO, and SO Groups. **(D)** UMAP plot revealed distribution of Expression for 8 Key Genes (SYK, TYROBP, ACTB, etc.) in All Cells of ATM.**E.** Percentage of Each Cell Type in HD, MO, and SO Groups.

Based on cell distribution, no significant changes in cell types were observed across adipose tissues at different BMI levels. However, there was noticeable heterogeneity in the composition ratios of cell types among various adipose tissues (**Figures 3B, F**). Interestingly, compared to the HD group, the MO and SO groups showed a significant increase in fibroblast proportions, indicating a potential BMI correlation. In contrast, adipocyte proportions decreased linearly with increasing BMI. The MO group also exhibited a notably higher proportion of T cells. Macrophages were evenly distributed across adipose tissues at different BMI levels, with slightly higher levels observed in the HD group.

The expression patterns of key genes associated with BMI in ATM were investigated: SYK, CD86, CSF1R, HCK, TYROBP, and LAPTM5 were predominantly expressed in macrophages, whereas ITGB2 showed primary expression in T cells (**Figures 3C, D**; **Supplementary Figure S2D**). This highlights the pivotal role of the interaction between macrophages and adipocytes in shaping obesity, emphasizing the significant regulatory function of the immune system in obesity development (27).

### 3.3 Macrophage subtypes heterogeneity in ATM across varying BMI levels

**Figure 4:**
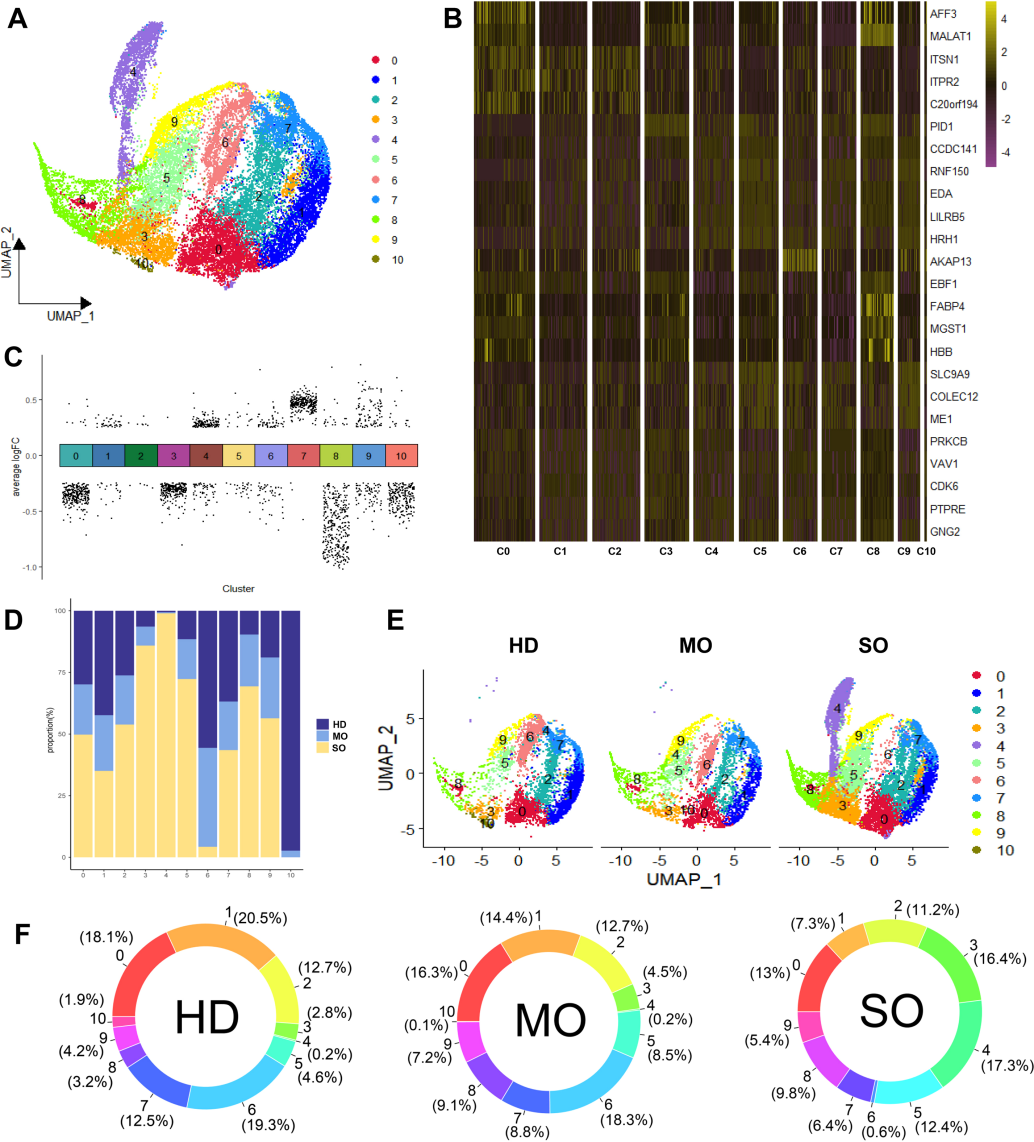
Panoramic landscape of macrophages in ATM. **(A)** UMAP plot revealed the distribution of 11 Macrophage Subtypes Based on NCM. **(B)** Heatmap of FDGs in Each Macrophage Subtype. For each cluster the top 5 genes and their relative degree levels in all Macrophage cells are shown. **(C)** DDGs Results for Each Subtype. **(D)** Percentage of HD, MO, and SO Groups in Each Macrophage Subtype. **(E)** UMAP plot revealed cellular heterogeneity with 11subtypes of cells identified in HD, MO, and SO Groups and color-coded. **(F)** Percentage of Each Macrophage Subtype in HD, MO, and SO Groups. **(G)** GO Enrichment Analysis of Each Macrophage Subtype. Red squares indicate enrichment of the biological process, while white squares indicate no enrichment of the biological process.

We examined how BMI levels affect the roles and distribution of macrophage types in adipose tissue. BMI-related genes were predominantly expressed in macrophages. Using the CSN algorithm, we analyzed 23,681 macrophages from the scRNA-seq dataset and constructed a gene regulatory network for each cell. Later on, we identified 11 distinct macrophage subtypes (**Figure 4A**) and assessed their relative contributions across different BMI groups (**Figure 4F**).

In the HD group, predominant macrophage subtypes included C1 (20.5%) and C6 (19.3%). MO group exhibited higher levels of C6 (18.3%) and C0 (16.3%), whereas SO group showed increased proportions of C4 (17.3%) and C3 (16.3%). C6 was prominently present in both HD and MO groups, distinguishing them from the SO group. Specifically, C10 was predominant in HD, while C4 was more prevalent in SO. Notably, the MO group showed significantly elevated levels of C6, indicative of its association with MO (**Figure 4D**).

To identify regulatory drivers among the 11 subtypes, we defined genes with high degree and discriminative power (logFC > 0.5, AUC > 0.75) within each subtype as FDGs. Most FDGs were unique to individual subtypes, with only a few shared across multiple subtypes. For example, AKAP13, specific to C6, showed minimal expression in other macrophage subtypes (**Figure 4B**). Notably, specific markers for subtypes C4 and C10 were not found, suggesting differences between these subtypes are likely functional rather than gene marker distinctions. Despite limitations in gene marker-based assessments for macrophages, we used the Wilcoxon test algorithm to identify subtype-specific DDGs and conducted GO enrichment analysis to annotate primary biological processes associated with each subtype (**Figures 4C, G**).

C0: 9 upregulated DDGs (e.g., ITSN1, ITPR2, C20orf194) and 288 downregulated DDGs (e.g., HRH1, ATRN, RNF150), with no notable biological process enrichment.
C1: 38 upregulated DDGs (e.g., FGD4, DMXL2, ADK) and 21 downregulated DDGs (e.g., ANKUB1, AC010127.1, AP003086.2), involved in cell morphogenesis, maintenance, and protein metabolism.
C2: 7 upregulated DDGs (e.g., C20orf194, ITSN1, FGD4) and 5 downregulated DDGs (e.g., HRH1, MALAT1, ABCA9), contributing to transmembrane substance transport and telomere maintenance regulation.
C3: 1 upregulated DDG (PID1) and 256 downregulated DDGs (e.g., SNX24, EPB41L2, PTPRM), associated with insulin response regulation and monosaccharide transmembrane transport.
C4: 136 upregulated DDGs (e.g., CFD, RCSD1, LUC7L3) and 54 downregulated DDGs (e.g., ZFPM2, BNC2, PLCB1), involved in macrophage activation, inflammation, and phosphatase activation.
C5: 15 upregulated DDGs (e.g., RNF150, HRH1, MAMDC2) and 29 downregulated DDGs (e.g., ITSN1, DOCK10, ITPR2), playing roles in tissue and organ development and differentiation.
C6: 57 upregulated DDGs (e.g., AKAP13, MAP3K1, SNX24) and 44 downregulated DDGs (e.g., FOS, LMNA, NAMPT), involved in proliferation, migration, chemotaxis, and cell adhesion across various cell types.
C7: 301 upregulated DDGs (e.g., CELF2, PNISR, DDX17) and 3 downregulated DDGs (e.g., MALAT1, FABP4, HBB), influencing cell-matrix interactions, cell size regulation, and responses to extracellular signals.
C8: 20 upregulated DDGs (e.g., MALAT1, ZBTB20, FABP4) and 331 downregulated DDGs (e.g., HNRNPC, PPP1R12A, ZNF638), without significant biological process enrichment observed.
C9: 97 upregulated DDGs (e.g., AUTS2, ARHGAP18, ME1) and 75 downregulated DDGs (e.g., ITSN1, PRKCB, RIPOR2), without significant enrichment of biological processes observed.
C10: 16 upregulated DDGs (e.g., AKAP13, CELF2, ZEB2) and 220 downregulated DDGs (e.g., RAB31, MITF, RNF149), involved in insulin-stimulated response, transmembrane transport, and immune response activation.

These findings highlight distinct gene regulatory patterns and functional roles of macrophage subtypes in adipose tissue. Macrophages in ATM are crucial for cell development, tissue growth, immune response, and energy regulation. Specifically, subtypes C4, C6, and C10 exhibit unique functions related to obesity. C10 supports energy metabolism, immune balance, insulin response, and glucose transport. C6 regulates macrophage chemotaxis, migration, and helps maintain adipose tissue balance during obesity. Conversely, activated C4 may exacerbate obesity by triggering inflammation and peptide tyrosine kinase activation. In the HD group, C10 functions independently, while in obesity, C6 and C4 reflect the body’s regulatory responses, contributing to inflammation and exacerbation of obesity. Notably, C6 and C4 likely represent different stages of differentiation among obesity-related macrophages.

### 3.4 Exploring macrophage differentiation patterns across BMI variations

**Figure 5:**
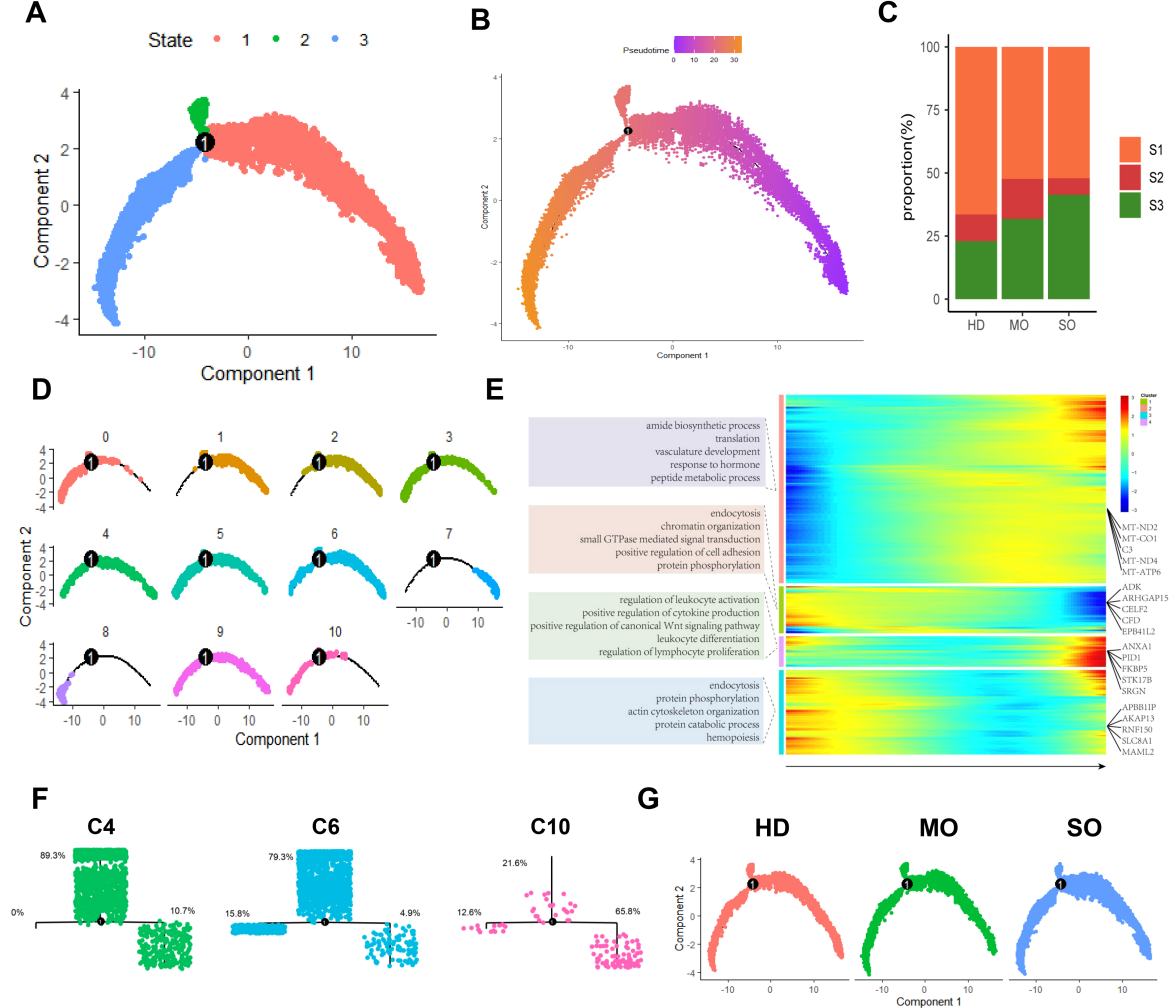
Macrophage differentiation patterns in ATM across different obese levels. **(A)** The Macrophage trajectory predicted by monocle 2. **(B)** Pseudotime trajectory of Macrophages with pseudotime. **(C)** Percentage of HD, MO, and SO Groups in Each State. **(D)** The differentiation trajectories of distinct macrophage subtypes. **(E)** Heatmap of the degree levels for all DEGs in all macrophages. **(F)** Differentiation Trajectories and Distribution of States in Macrophage Subtypes C4, C6, and C10. **(G)** Differentiation Trajectories of HD, MO, and SO groups.

The scRNA-seq analysis is a powerful tool for simulating and uncovering cell differentiation processes, enhancing our understanding of cellular development. Using the Monocle2 algorithm, we mapped macrophage differentiation trajectories across varying BMI populations based on macrophage NCM (**Figures 5A, B**) (28). Biological processes result delineated two distinct stages of macrophage differentiation: (a) Early differentiation states (S1, S2) are characterized by regulatory processes in cell morphogenesis (e.g., cytoskeleton organization, cell morphogenesis) and signal transduction pathways (e.g., small GTPase-mediated signal transduction, enzyme-linked receptor protein signaling pathway). (b) Advanced differentiation states (S3) feature processes including peptide metabolism (e.g., peptide biosynthetic process, peptide metabolic process), angiogenesis (e.g., blood vessel development, vasculature development), and immune cell functions (e.g., positive regulation of immune response, regulation of lymphocyte activation) (**Figure 5E**).

We correlated macrophage differentiation states (S1, S2, S3) with BMI groups. We observed an increase in the higher differentiation state S3 as obesity severity rose, reaching a peak of 41.3% in the SO group. On the other hand, the lower differentiation state S1 decreased with increasing obesity (**Figures 5C, G**). This suggests that bone marrow-derived mononuclear cells differentiate into tissue-specific macrophages in adipose tissue under the influence of the local microenvironment. These dynamics vary with obesity, contributing to diverse morphologies and phenotypes.

We observed significant diversity in their differentiation states (**Figure 5D**). Specifically, C10 tends to exhibit a higher differentiation state, with the majority of cells in the S3 state (C10: 65.8%). In contrast, C6 and C4 generally show lower differentiation states, with most cells in the S1 state (C6: 79.3%, C4: 89.3%). These findings suggest that obesity-induced ATM reprograms macrophage differentiation (**Figure 5F**).

### 3.5 The distinctive gene regulatory patterns exhibited by specific macrophage subtypes in different BMI groups

**Figure 6:**
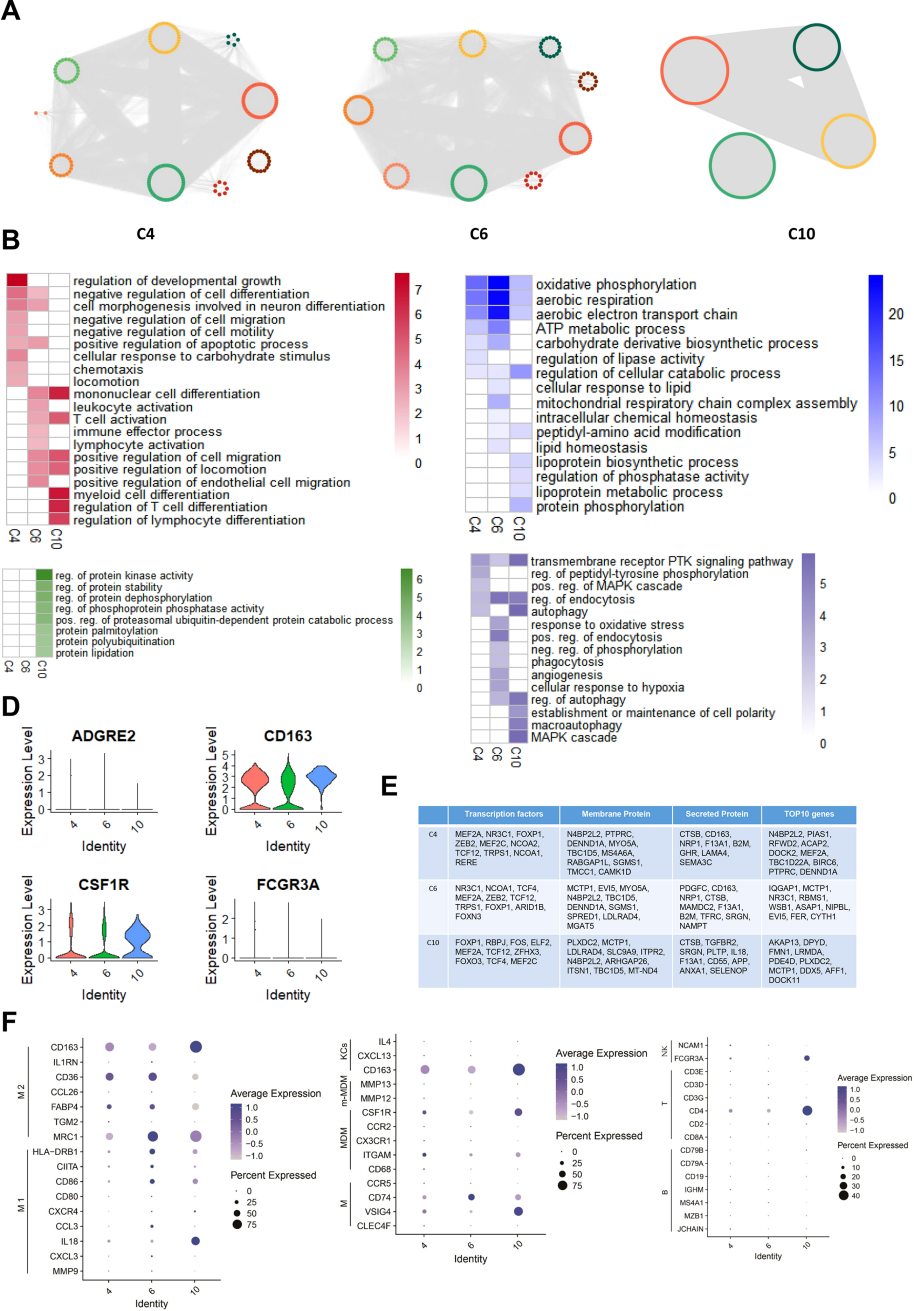
ssGRN Analysis of specific macrophage subtypes in ATM Across Different Obese levels. **(A)** Identifying gene modules based on Macrophage Subtypes ssGRNs C4, C6, and C10. For each subtype’s ssGRN, we identified gene modules using DeepWalk combined with GML algorithm. **(B)** GO Enrichment Analysis for Regulatory Networks of Macrophage Subtypes C4, C6, and C10. In the figure, red represents immune response processes, green represents protein modification processes, blue represents metabolic processes, and purple represents other processes. The heatmap values indicate the maximum -log P value of each term across all gene modules in the respective cell subtype. **(C)** Distribution of Macrophage Markers CD163, FCGR3A (CD16), ADGRE2 (CD312), and CSF1R (CD315) across three Macrophage subtypes. **(D)** ssGRNs of Macrophage Subtypes C4, C6, and C10: Top 10 Genes, Transcription Factors, Secreted Proteins, and Membrane Proteins Based on Degree. **(E)** The Bubble Charts illustrate the expression distribution of markers for M1 macrophages, M2 macrophages, Kupffer cells (KCs), macrophages, monocyte-derived macrophages (MDM), m-MDM cells, NK cells, B cells, and T cells within subtypes C4, C6, and C10, respectively.

We constructed subtype-specific gene regulatory networks (ssGRNs) for C4, C6, and C10, analyzing them with the Deepwalk and Gaussian Mixture model cluster algorithms to identify gene modules. Our findings revealed 10 gene modules in C4, 9 in C6, and 4 in C10 (**Figure 6A**). Subsequently, we performed GO enrichment analysis on each module, demonstrating significant involvement in immune responses and metabolic processes across all three subtypes. Notably, the C10 subtype is significantly enriched in protein modification-related biological processes. While all three subtypes participate in immune responses and metabolism, their specific biological activations differ. C4 primarily induces processes related to cell shape changes, including negative regulation of cell differentiation, neuron differentiation-related morphogenesis, and apoptosis. It also activates lipid metabolism processes. In contrast, C6 primarily activates T cell-related immune cell activation and chemotaxis, alongside energy and lipid metabolism processes, particularly mitochondrial oxidative phosphorylation. C10 primarily triggers biological processes related to myeloid lymphocyte differentiation and lipoprotein metabolism. Furthermore, both C4 and C10 activate MAPK signaling pathways and autophagy. C6 specifically induces oxidative stress and phagocytosis, whereas C10 specifically activates processes related to establishing and maintaining cell polarity. (**Figure 6B**).

Transcription factors, membrane proteins, and secretory proteins are pivotal for intracellular and intercellular signal transduction. We identified the top 10 genes with the highest Degree values in gene regulatory networks for each macrophage subtype, encompassing these categories. While similarities exist among driver genes and regulatory proteins across C4, C6, and C10 subtypes, notable differences also distinguish each subtype. Remarkably, C4 and C6, specific to obese populations, exhibit significant overlaps in key transcription factors (e.g., MEF2A, NR3C1), membrane proteins (e.g., N4BP2L2, DENND1A), and secretory proteins (e.g., CD163, NRP1) (**Figure 6D**). In contrast, C10, specific to HD populations, displays more independent communication functions (**Figure 6B**), underscoring the heterogeneous nature of macrophage subtypes and their differentiation patterns. Under healthy conditions, C10 maintains a distinct regulatory pattern, while C6 and C4 are shaped by the obese tissue environment, showing similar regulatory mechanisms and functions associated with obesity. However, their differentiation statuses vary with the severity of obesity.

We validated cellular functions through gene expression level, confirming the macrophage identity of the three subtypes by measuring markers such as CD163, FCGR3A/CD16, ADGRE2/CD312, and CSF1R/CD315 (**Figure 6C**). Macrophages exhibit distinct activation states M1 and M2 each with specific roles in immune responses (29). Our analysis showed that C4, C6, and C10 predominantly express M2 macrophage markers like MRC1 (CD206), FABP4, CD36, and CD163, with C6 and C10 also expressing M1 markers (**Figure 6E**). This suggests that C4, C6, and C10 exhibit characteristics of M2 macrophages, potentially contributing to anti-inflammatory responses in adipose tissue.

Furthermore, we compared marker gene expression between these subtypes and macrophages from various tissues. C4 and C6 displayed similarities with typical liver macrophages (expressing CD74, VSIG4) and Kupffer cells (expressing CD163). In contrast, C10 exhibited elevated expression of VSIG4 (typical liver macrophage marker), CSF1R (mature monocyte-derived macrophage marker from liver), and CD163 (Kupffer cell marker) (**Figure 6E**).

This suggests that macrophage subtypes C4, C6, and C10 may originate from liver tissue, potentially recruited to adipocytes via specific molecules. Additionally, we compared their expression profiles with T cells, B cells, and NK cells (30–32). Subtype C10 showed expression similarities with markers found in CD4+ T cells and NK cells (**Figure 6E**), indicating potential shared biological functions.

**Figure 7:**
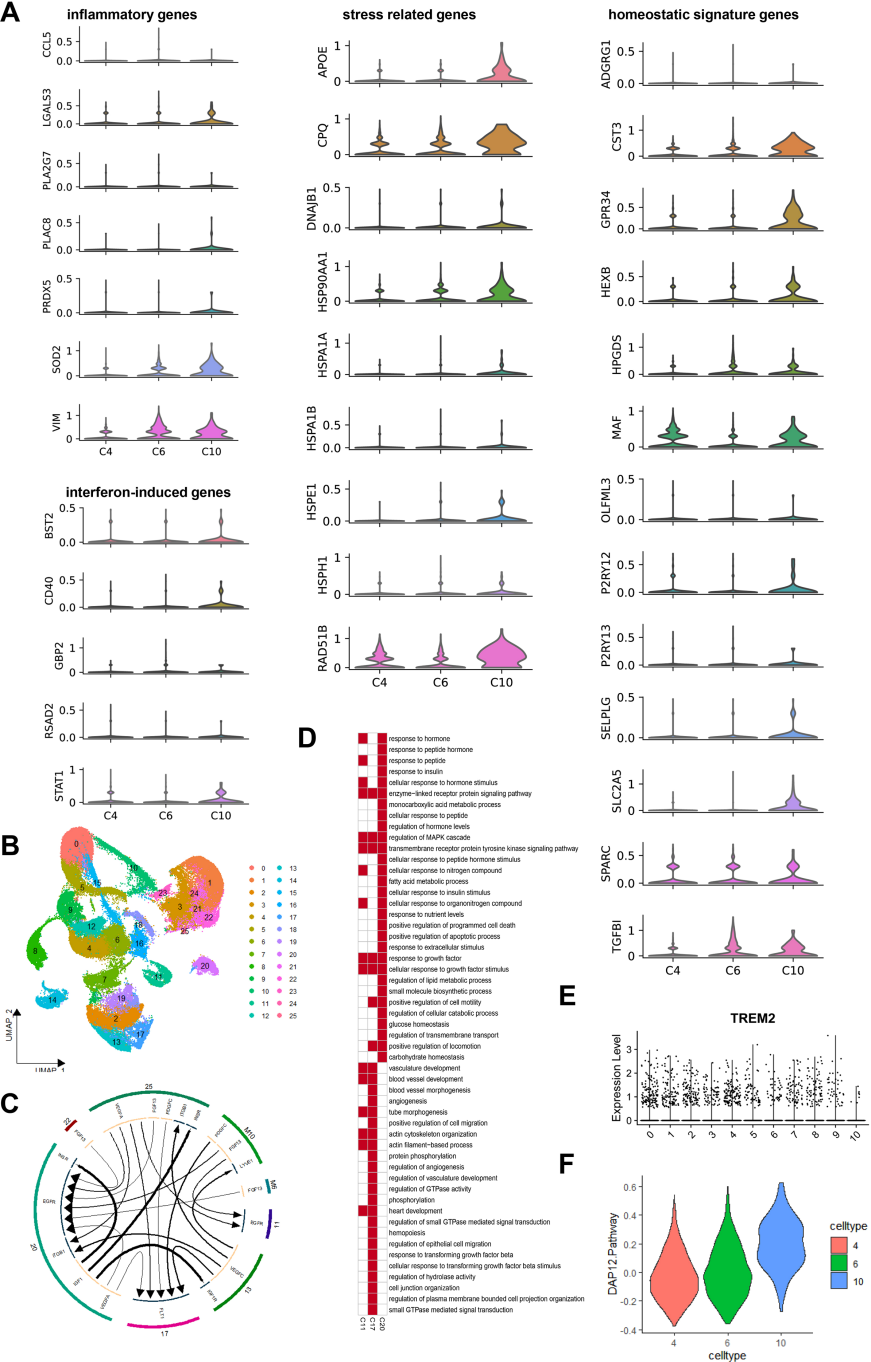
B Function analysis of macrophage subtypes C4, C6, and C10 and cell communication with adipocytes. **(A)** Inflammatory Genes Expression Distribution (violin plot) in Macrophage Subtypes C4, C6, and C10. **(B)** Stress Related Genes Expression Distribution (violin plot) in Macrophage Subtypes C4, C6, and C10. **(C)** Homeostatic Signature Genes Expression Distribution (violin plot) in Macrophage Subtypes C4, C6, and C10. **(D)** Interferon-Induced Genes Expression Distribution (violin plot) in Macrophage Subtypes C4, C6, and C10. **(E)** UMAP plot revealed that the distribution of Adipocyte Subtypes in Different Groups’ ATM. **(F)** Adipocyte subtypes and macrophage cellular communication outcomes are depicted in the figure, showcasing the dynamic interplay within adipose tissue. **(G)** GO Enrichment analysis results for adipocyte subtypes C11, C17, and C20. Red one indicates enrichment of the GO term within the subtypes, while white one indicates non-enrichment of the GO term within the subtypes. **(H)** Expression Distribution of Trem2 Gene Across All Macrophage Subtypes. **(I)** Activation Level Distribution of DAP12 Signaling Pathway Across Macrophage Subtypes C4, C6, and C10 used by ssGSEA Algorithm.

We assessed gene expression across four functional categories: inflammatory responses, interferon-induced regulatory processes, stress responses, and homeostatic signatures (33). Subtype C10 showed significant activation in inflammation, stress responses, and homeostasis regulation compared to C6 and C4. However, none of the subtypes displayed significant activation in interferon-mediated regulatory processes (**Figures 7A–D**).

Recent studies emphasize TREM2’s role in regulating adipose metabolism and macrophage survival (34, 35). TREM2 forms a complex with DAP12 to activate SYK downstream (36). Given that DAP12 and SYK are crucial genes correlating with BMI in adipose tissue, we analyzed TREM2 expression and DAP12 signaling pathway enrichment score across C4, C6, and C10 subtypes. Interestingly, we observed inhibited DAP12 signaling in C4 and C6 but activation in C10, despite higher TREM2 expression in C4 and C6 compared to C10 (**Figures 7H, I**). This suggests that worsening obesity suppresses the DAP12 signaling pathway in macrophages, potentially reducing their surveillance over adipocytes and contributing to obesity development.

### 3.6 Specific macrophage subtypes exhibit cell-to-cell communication with adipocytes

We used the iTalk algorithm to analyze cell communication among C4, C6, C10, and adipocytes in adipose tissue (**Figure 7F**). C10 macrophages interacted with adipocyte subtypes C11, C17, and C20, while C6 macrophages regulated adipocyte subtype C20. However, C4 macrophages showed minimal regulatory effects on adipocytes, indicating reduced impact in severely obese adipose tissue.

C10 macrophages targeted adipocyte subtypes C11, C17, and C20 through PDGFC-FLT1 and FGF13-EGFR signaling, while C6 macrophages influenced adipocyte C20 via FGF13-EGFR signaling. Using the Wilcoxon algorithm, we identified differentially expressed genes in adipocyte subtypes C11, C17, and C20 (|logFC| > 0.25 & P < 0.05) and conducted GO enrichment analysis (**Figures 7F, G**).

The adipocyte subtype C17 activated angiogenesis, TGF-β signaling, and epithelial cell migration, while the adipocyte subtype C20 was involved in insulin response, lipid metabolism, and growth factor stimulation. Notably, obesity-specific macrophages regulated hormone-mediated responses in adipocytes through targeted EGFR signaling (**Figures 7F, G**).

### 3.7 Drug therapy reduces high-fat diet model mice adipose load by regulating the DAP12-SYK signaling pathway

**Figure 8:**
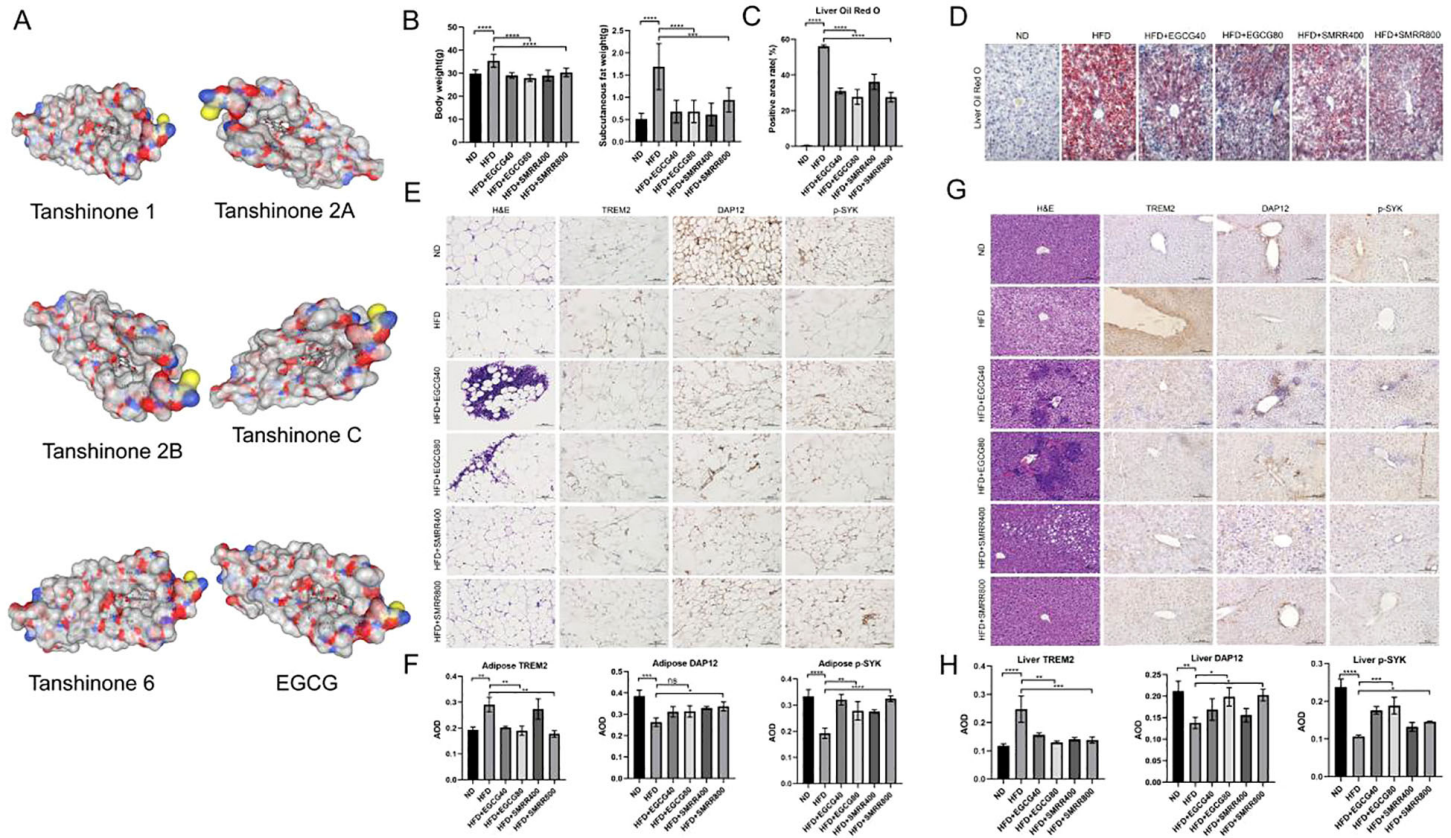
EGCG and SMRR reduces fat accumulation in HFD-fed mice by regulating DAP12-SYK signaling pathway. **(A)** The molecular docking results of DAP12 with EGCG and SSMR (Tanshinone 1, Tanshinone 2A, Tanshinone 2B, Tanshinone C, Tanshinone 6 and EGCG). **(B)** The effects of EGCG and SMRR treatment on body weight and subcutaneous fat weight. **(C)** Liver lipid content was assessed using Oil Red O staining (scale bar = 100 μm). **(D)** EGCG and SMRR attenuated the accumulation of HFD-fed mice’s lipid in the liver. **(E)** Subcutaneous fat size was assessed using H&E staining (scale bar = 100 μm). **(F)** The TREM2 expression of HFD-fed mice’s subcutaneous fat tissue was attenuated by EGCG and SMRR. DAP12, and p-SYK expression of subcutaneous fat tissue were enhanced by EGCG and SMRR. **(G)** liver lipid content was assessed using H&E staining (scale bar = 100 μm). **(H)** The TREM2 expression of HFD-fed mice’s liver were attenuated by EGCG and SMRR. DAP12, and p-SYK expression of HFD-fed mice’s subcutaneous fat tissue were enhanced by EGCG and SMRR. The results were presented as the average of the measurements in each group (Mean ± SD). *P<0.05, **P<.01, ***P<.001, ****P<.0001. ns, not significant.

Transcriptomic and scRNA analyses indicate that the obese microenvironment can activate specific macrophage subtypes through the DAP12-SYK pathway, thereby contributing to obesity. We found through molecular docking that various monomers of EGCG (EGCG-DAP12 (vina=-7.2), EGCG-SYK (vina=-7.8)) and SSMR (Tanshinone 1-DAP12 (vina=-8.0), Tanshinone 2A-DAP12 (vina=-8.5), Tanshinone 2B-DAP12 (vina=-8.5), Tanshinone C-DAP12 (vina=-8.4), Tanshinone 6-DAP12(vina=-7.5)) strongly bind to the DAP12-SYK signaling pathway (**Figure 8A**, **Supplementary Figure S1**). Studies also suggest that EGCG and SMRR may mitigate obesity-related complications. Therefore, EGCG and SMRR have been chosen for further research as potential drugs for treating obesity.

We induced obesity in mice with an 8-week high-fat diet (HFD). Compared to the normal diet (ND) group, HFD-fed mice showed significant increases in body weight and subcutaneous fat, Treatment with EGCG and SMRR markedly reduced body weight and fat mass in HFD-fed mice (**Figure 8B**), along with liver lipid deposition and fat degeneration. Liver fat degeneration improved, as evidenced by reduced Oil Red O staining and decreased lipid accumulation (**Figures 8C, D**). Adipocyte size in the treatment group was also notably smaller than in the model group, indicating an improvement in obesity progression.

Immunohistochemical analysis revealed increased TREM2 expression in the liver and subcutaneous adipose tissues of HFD-fed mice compared to the ND group. Meanwhile, levels of downstream DAP12 and p-SYK expression were reduced (**Figures 8E–H**). This suggests that in obesity, adipose tissue-specific macrophages are recruited and activated, but the downstream DAP12-SYK pathway is selectively inhibited, compromising its anti-obesity effects.

We treated HFD-fed mice with EGCG and SMRR, resulting in a significant reduction of TREM2 expression in liver and adipose tissue macrophages. Additionally, both EGCG and SMRR reversed the suppressed expression of the DAP12-SYK signaling pathway in obese mice, indicating their potential to counteract obesity induced by a high-fat diet. These results align with previous findings highlighting DAP12 enrichment in obesity-specific adipose tissue macrophages.

## 4 Discussion

In this study, we identified a gene module strongly associated with BMI by comparing adipose tissue transcriptomes of healthy and obese individuals. This module primarily regulates immunity and includes key genes like SYK and TYROBP. Using single-cell transcriptome analysis of adipose tissues, we found these genes are predominantly expressed in macrophages. We identified distinct macrophage subtypes exclusive to HD, MO, and SO groups, investigating their differentiation patterns, gene regulation, biological functions, and interactions with adipocytes. HD-specific subtypes operated somewhat independently, while those specific to obesity exhibited functions correlated with changes in BMI. As obesity progressed, macrophage regulation of adipocytes diminished, particularly evident in the SO group where the C4 subtype ceased its regulatory role. This suggests adipose tissue macrophages may adapt to promote obesity. The DAP12-SYK signaling pathway emerged as crucial for macrophages in controlling adipocyte lipid accumulation. Analysis revealed varying DAP12 signaling activation levels across obesity stages, declining with severity. *In vivo*, SMRR and EGCG activated DAP12-SYK, mitigating adipocyte lipid accumulation and preventing obesity. These findings shed light on obesity’s molecular mechanisms and potential therapeutic avenues.

Transcriptomic analysis revealed 8 genes linked to BMI: SYK, CD86, CSF1R, HCK, TYROBP (DAP12), LAPTM5, ITGB2, and ACTB. SYK is pivotal in macrophage-driven inflammation, crucial in responding to oxidized low-density lipoprotein via autophagy, regulating immune responses and inflammation (37). CD86, an immunoglobulin superfamily member, stimulates T cell activation via CD28 binding and negatively regulates it via CTLA-4 (38). CSF1R controls macrophage differentiation and growth (39, 40). HCK, a Src family kinase involved in matrix adhesion and tissue degradation via podosomes, affects bone marrow-derived macrophages’ 3D migration and matrix protein degradation capabilities, hindering their interstitial tissue migration (41). Increased HCK expression is linked to inflammation and fibrosis. Inhibiting HCK reduces pro-inflammatory M1 macrophage polarization and promotes anti-inflammatory M2 polarization, crucial in kidney injury and as a therapeutic target for reducing renal inflammation and fibrosis (42). DAP12 regulates immune responses by modulating receptors on macrophages and other immune cells, activating macrophages and regulating inflammation (43). DAP12-SYK signaling is essential for cytokine-induced macrophage fusion, enhancing this process in granulomatous inflammation (44). LAPTM5 activates NF-κB and MAPK pathways in macrophages, promoting cytokine release and inflammation (45). ITGB2 participates in immune activities related to glioblastoma, influencing B cells, CD4+ T cells, macrophages, neutrophils, and dendritic cells, and plays roles in energy metabolism, cell cycle regulation, angiogenesis, and neuronal myelin formation and repair after ischemic stroke (46). ACTB encodes β-actin, essential for cell cytoskeleton integrity, impacting cell invasion and metastasis. These genes play critical roles in obesity development (47). The close association of these genes suggests they may play key roles in the development of obesity.

At the single-cell level, we explored the diversity of ATM across different BMI groups, revealing a significant presence of immune cells. T cells were notably more abundant in the adipose tissues of individuals with MO compared to those with HD and SO groups. Meanwhile, macrophages were consistently present across all BMI populations.

This aligns with previous studies indicating that obesity triggers chronic inflammation, impacting the body’s immune response (48, 49). Dam V et al. identified T cells and macrophages as the main immune cells in adipose tissue (50). Key BMI-associated genes in module M2, like SYK, CD86, CSF1R, HCK, TYROBP, and LAPTM5, are predominantly expressed in macrophages. This underscores the crucial role of macrophages in regulating processes within obese adipose tissue, potentially influencing obesity’s development and progression.

This study employs an analytical method to identify macrophage subtypes in different BMI populations based on gene centrality within cellular regulatory networks. It identifies the presence of macrophage subtype C10 in HD, C6 in MO, and C4 in SO groups, all primarily involved in immune responses and metabolic processes. C10 subtype demonstrates specificity in protein modification processes and post-translational modifications (e.g., acylation, sumoylation, phosphorylation), crucial for fat absorption, obesity-induced inflammation, and metabolic disorders (51–53). This suggests that C10 subtype found in healthy tissues may influence obesity. Furthermore, comparing these three macrophage types with traditional M1 and M2 macrophages reveals a closer resemblance to M2 macrophages, characterized by CD206 (MRC1) expression. In HD group, M2 macrophage markers increase, but decrease in obese group, indicating macrophage reprogramming towards a potential shift from M2-like to M1-like phenotypes, consistent with Lumeng et al. (54). Moreover, assessing inflammatory, stress, homeostasis, and interferon-mediated responses among these cell types shows that the C10 subtype, compared to obesity-specific C4 and C6 subtypes, exhibits beneficial anti-inflammatory and homeostasis-maintaining functions. This suggests that the obese ATM may compromise macrophage immune surveillance, fostering conditions conducive to obesity development.

Cell communication studies show that macrophages not only reduce lipid droplet formation but also directly influence adipocytes via the FGF13-EGFR pathway, regulating insulin secretion and angiogenesis in adipose tissue. Previous research indicates that M1-like inflammatory macrophages can affect angiogenesis gene expression in preadipocytes (55, 56). Additionally, Cao et al. reveals that a high-fat diet (HFD) increases EGFR and its ligands in adipose tissue macrophages. Selective loss of EGFR in adipose tissue macrophages inhibits their proliferation and monocyte infiltration into adipose tissue, thereby reducing obesity and insulin resistance (57). These findings provide insights into how chronic inflammation in adipose tissue contributes to insulin resistance in obesity.

DAP12 signaling pathway plays a crucial role in enhancing ATM (43, 44, 58). Our findings show that TREM2+ macrophages are recruited to adipocytes, inhibiting lipid droplet formation (34). However, in obesity, there’s a gradient decrease in the activation of the downstream DAP12-SYK signaling pathway in activated macrophages. Activating the TREM2-mediated DAP12-SYK pathway in ATMs could potentially suppress the onset of obesity. Jaitin et al. identified a novel TREM2+ lipid-associated macrophage subset in adipose tissue and demonstrated that deletion of the TREM2 gene inhibits this program, leading to adipocyte hypertrophy, hypercholesterolemia, increased body fat, and glucose intolerance (34). Recent studies have shown that activating the TREM2/DAP12 pathway, either directly (with a TREM2 antibody) or indirectly (with M-CSF), enhances SYK gene activity in brain microglia, mitigating responses to high-calorie, HFD-fed diets (59–62). Thus, targeting the TREM2-DAP12-SYK pathway in macrophages not only affects lipid droplet formation in adipose tissue but also modulates the body’s response to HFDs, potentially mitigating obesity progression at its core.

Both EGCG, a predominant catechin in green tea with proven benefits in cancer, diabetes, and cardiovascular diseases (63), and SMRR, known for its therapeutic effects in heart diseases and acute myeloid leukemia, exhibit pleiotropic antioxidant and anti-inflammatory activities (64, 65). To evaluate their specificity toward the TREM2–DAP12–SYK pathway, we integrated transcriptomic and single-cell analyses with molecular docking predictions. Our data indicate that the obese microenvironment activates distinct macrophage subtypes via the DAP12–SYK axis, contributing to metabolic dysfunction. Docking analyses further revealed strong binding affinities of EGCG (EGCG–DAP12: –7.2; EGCG–SYK: –7.8) and multiple SMRR monomers (e.g., Tanshinone 1–DAP12: –8.0; Tanshinone 2A–DAP12: –8.5) to components of this pathway, supporting a direct interaction with DAP12–SYK signaling. Combined with prior evidence that EGCG and SMRR ameliorate obesity-associated inflammation and lipid accumulation, these findings suggest that their anti-obesity effects may be mediated, at least in part, through modulation of the DAP12–SYK pathway in adipose tissue macrophages. Nonetheless, other signaling cascades such as NF-κB or MAPK may also contribute and warrant further investigation (66, 67).

Furthermore, we observed an intriguing correlation: gene module M2, strongly linked to BMI, predominantly resides on chromosome 19. Recent genome-wide association studies (GWAS) focusing on adipose tissue have identified a locus on chromosome 19 significantly associated with spontaneous fat breakdown (68). This suggests that alterations at the multi-omics level on chromosome 19 may closely correlate with obesity severity in organisms, potentially offering a pivotal avenue for future research and treatment of obesity. Our study of obese adipose tissue revealed a significant increase in T cell proportions in moderately obese group. Pathological sections further indicated substantial lymphocyte infiltration in the EGCG treatment group. Further investigation is needed to understand the involvement and regulatory role of T cells in obese adipose tissue.

We acknowledge several limitations in the current study. First, although our mouse experiments demonstrated the therapeutic efficacy and macrophage remodeling effect of the Natural Compounds EGCG and SMRR, comprehensive metabolic evaluations such as glucose tolerance, insulin resistance, and lipid profiling were not included. These parameters are essential to fully substantiate the systemic metabolic benefits and will be addressed in future studies. Second, the single-cell identification of macrophage subtypes (C4, C6, C10) was based primarily on computational clustering and transcriptomic signatures. Further experimental validation using flow cytometry, qPCR, and immunofluorescence to confirm subtype-specific markers and functions in adipose tissue macrophages will be necessary to consolidate these findings.

In summary, our research integrates transcriptome and single-cell sequencing technologies to uncover profound transcriptomic and microenvironmental distinctions between adipose tissues of healthy and obese individuals. We identified eight BMI-associated genes predominantly expressed in macrophages: SYK, CD86, CSF1R, HCK, TYROBP, LAPTM5, ITGB2, and ACTB. Furthermore, we identified distinct macrophage subtypes across different obesity levels and clarified their roles in adipocyte communication. *In vivo* experiments confirmed the pivotal role of the TREM2-DAP12-SYK pathway in obesity development, highlighting the efficacy of SMRR and EGCG in mitigating obesity through this pathway. This study advances our understanding of obesity pathogenesis, identifies potential therapeutic targets, and opens new avenues for innovation in obesity treatment and drug development.

## Data Availability

The original contributions presented in the study are included in the article/**Supplementary Material**, further inquiries can be directed to the corresponding author/s.

## Supplementary material

Supplementary Table 1 Cellmarker of all celltype in ATM.

Supplementary Figure 1 The molecular docking results of SYK with EGCG and SSMR (Tanshinone 1, Tanshinone 2A, Tanshinone 2B, Tanshinone C, Tanshinone 6 and EGCG).

Supplementary Figure 2 **(A)** The distribution of all genes from Functional Gene Module M2 on the human reference genome (hg19). **(B)** the network represent the PPI network of Functional Gene Module M2.Red nodes represent HUB genes in the PPI network, Blue nodes represent genes in the PPI network. **(C)** UMAP plot revealed cellular heterogeneity with 24 distinct clusters of cells identified and color-coded. General identity of each cell cluster is defined on the right. Parameter R refers to Seurat’s FindClusters function and determined clustering resolution. **(D)** Expression Distribution (violin plots) of 8 Key Genes (SYK, TYROBP, ACTB, etc.) Across Each Cell Type.
