# Supplementary material for: Macrophage activation of the TREM2-DAP12-SYK pathway shapes the adipose tissue microenvironment in obesity and unveils the therapeutic potential of natural compounds egcg and SMRR
Source: Front Immunol. 2025 Dec 11;16:1694985. doi: 10.3389/fimmu.2025.1694985 (PMC12739555; doi:10.3389/fimmu.2025.1694985)
Supplement: Supplementary Table 1 — Cellmarker of all celltype in ATM. [file DataSheet1.docx]

Table S1 Cellmarker of all celltype in ATM

| Macrophage | Fibroblast | T cell | Adipocyte |
| --- | --- | --- | --- |
| CD14 | CD111 | CD3 | CD34 |
| CD45 | CD140b | CD25 | CD36 |
| CD68 | CD156b | CD4 | CD31 |
| CD16 | CD271 | CD8 |  |
| CD206 | CD90 |  |  |
| CD32 | COL11A1 |  |  |
|  | GPR77 |  |  |


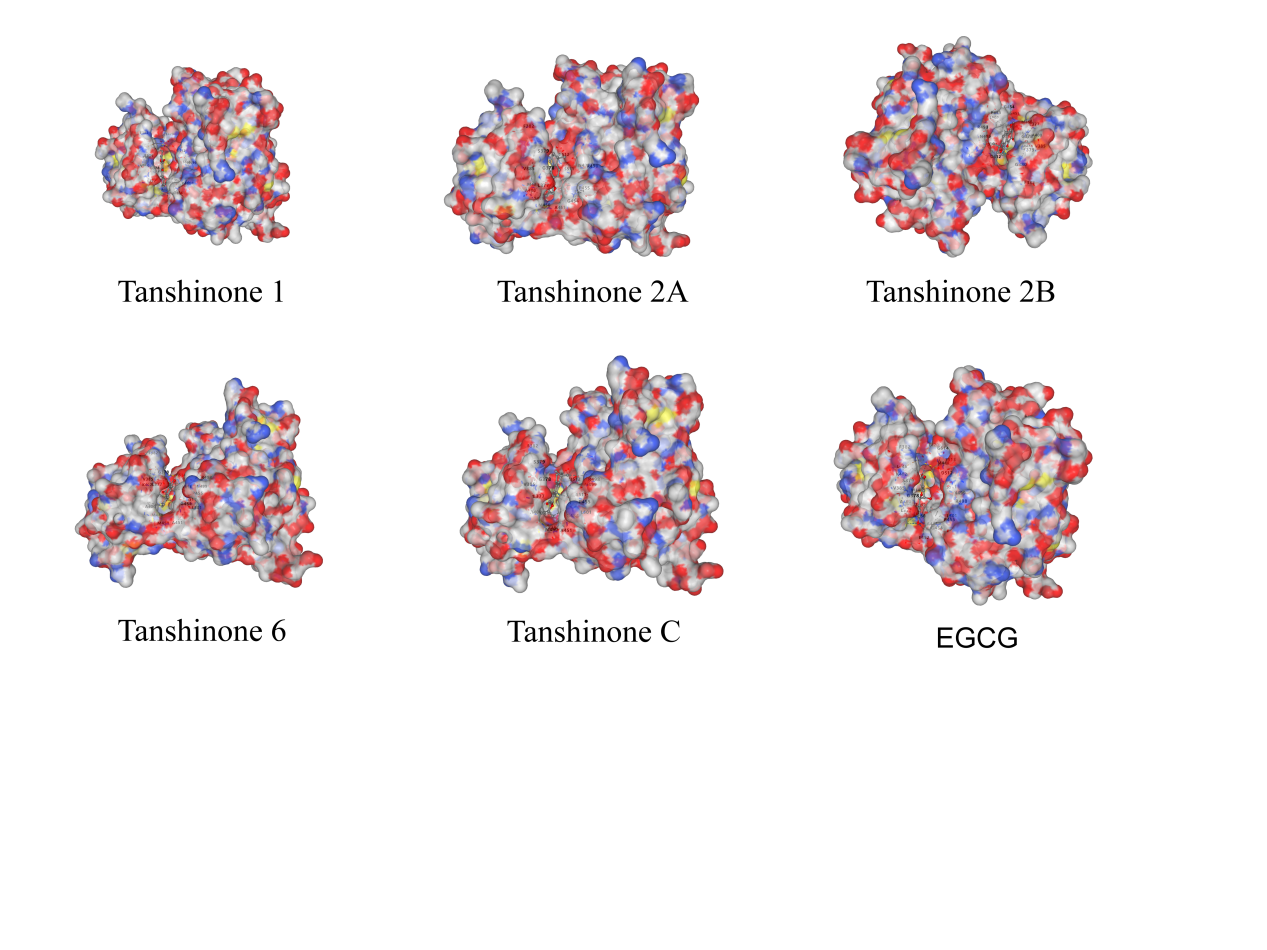


Fig S1 The molecular docking results of SYK with EGCG and SSMR (Tanshinone 1, Tanshinone 2A, Tanshinone 2B, Tanshinone C, Tanshinone 6 and EGCG).


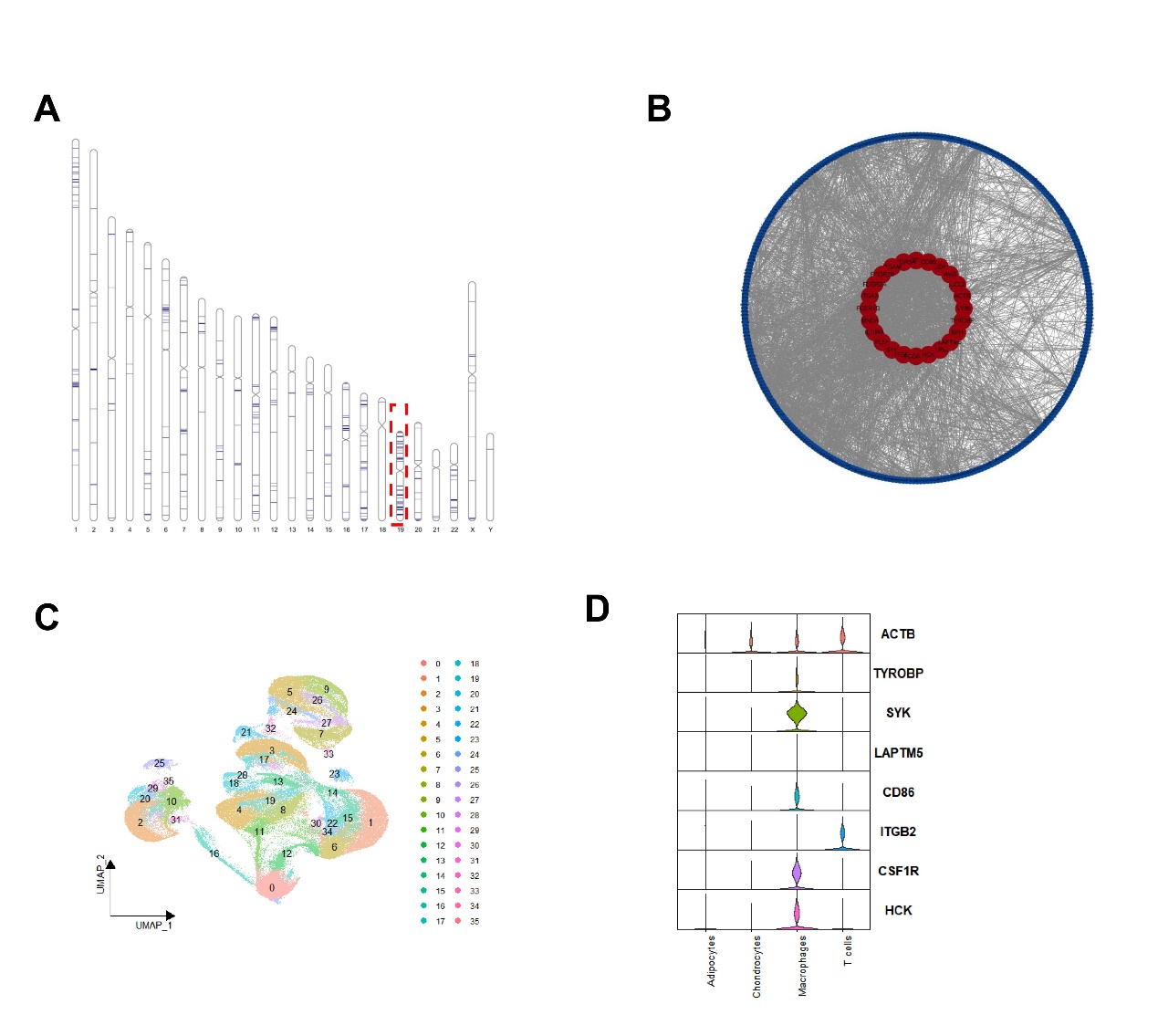


Figure S2 A. The distribution of all genes from Functional Gene Module M2 on the human reference genome (hg19). B. the network represent the PPI network of Functional Gene Module M2.Red nodes represent HUB genes in the PPI network, Blue nodes represent genes in the PPI network. C. UMAP plot revealed cellular heterogeneity with 24 distinct clusters of cells identified and color-coded. General identity of each cell cluster is defined on the right. Parameter R refers to Seurat’s FindClusters function and determined clustering resolution. D. Expression Distribution (violin plots) of 8 Key Genes (SYK, TYROBP, ACTB, etc.) Across Each Cell Type.
